# Closed reduction of a traumatic hip dislocation in children: case report

**DOI:** 10.11604/pamj.2017.26.231.12283

**Published:** 2017-04-25

**Authors:** Achraf El Bakkaly, Fouad Ettayebi, Houda Oubeja, Mounir Erraji, Hicham Zerhouni

**Affiliations:** 1 The Pediatric Surgical Emergencies Children’s Hospital CHU Rabat, University Mohammed V, Faculty of Medicine, Rabat, Morocco

**Keywords:** Hip dislocation, traumatic, child, closed reduction

## Abstract

Traumatic dislocation of the hip in children is a rare disease. It only represents 5% of hip dislocations in all age groups. Before 10 years, the mechanism is often a minimal domestic accident; after 10 years, the dislocation occurs with the waning of an accident of the public highway. It is different from that of the adult by its rarity, its ease of reduction and better prognosis. This is an emergency trauma: risk necrosis of the femoral head (If delayed reduction). We report a rare case of a 3 year old boy, who suffered from bipolar trauma after a fall near his height of his house causing him a detachment of the right humerus and post-traumatic dislocation of the left hip. The diagnosis was clinically confirmed by the results of standard radiographs and CT scans of the pelvis. The consultation period to emergencies was 5 hours after the trauma. We performed an hour after a closed reduction under general anesthesia for hip dislocation with establishment of a splint pelvic-pedal for analgesic keep for three weeks. The radiological outcome was satisfactory. Peeling Salter I humerus was reduced by orthopedic manner and immobilized by thoracoabdominal plaster to keep for a month. The child was discharged the next day. Reviewed in consultation after a month, the clinical examination showed a steady left hip. Traumatic dislocation of the hip in children is a rare diagnosis, the management should as urgent as possible to overcome the different possible subsequent complications dominated by coxa magna.

## Introduction

Traumatic dislocation of the hip in children is a rare disease, representing 2-5% of all dislocations in all age groups. The mechanism that is often not violent before 10 years, usually with the waning of a small domestic accident, becomes violent after 10 years since the dislocation occurs with the waning of an accident of the public highway [1]. It is a therapeutic emergency which requires a reduction of pure dislocation under anesthesia in good conditions because complications are not small, dominated by the risk of post-traumatic necrosis and long-term osteoarthritis [2]. Our observation shows this unusual injury, so little known, little codified care and whose series reported in the literature are modest in number of cases. We will proceed through the study of our case of traumatic dislocation of the hip in children to clarify the epidemiological, treatment of this disease and its long-term development with a review of published series.

## Patient and observation

**Figure 1:**
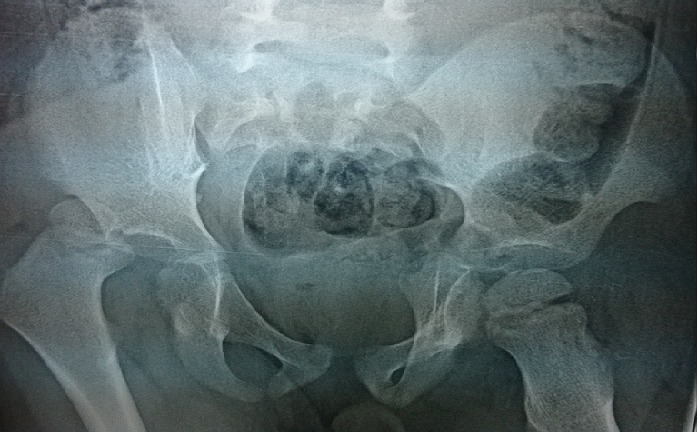
Standard radiography of the pelvis showing an aspect of a high pure right posterior iliac variety dislocation without fracture lesions

**Figure 2:**
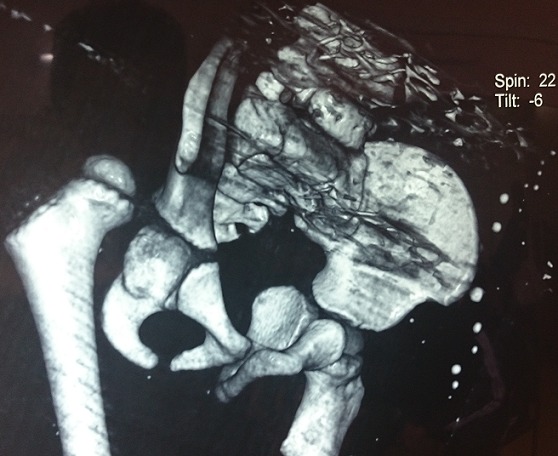
Scan Image reconstruction confirming the pure posterior iliac variety of dislocation without any associated fracture

**Figure 3:**
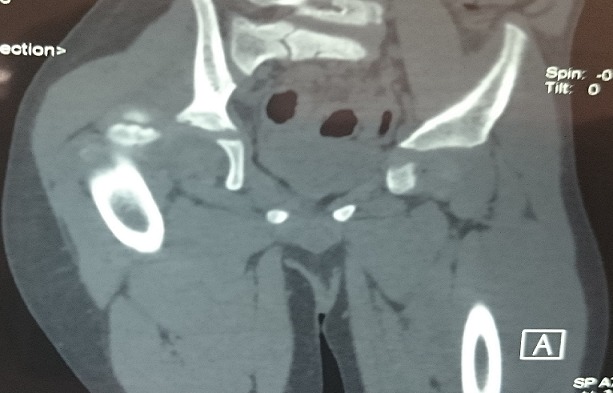
Scannographic cross section objectifying the sliding of the femoral head out of the acetabulum

**Figure 4:**
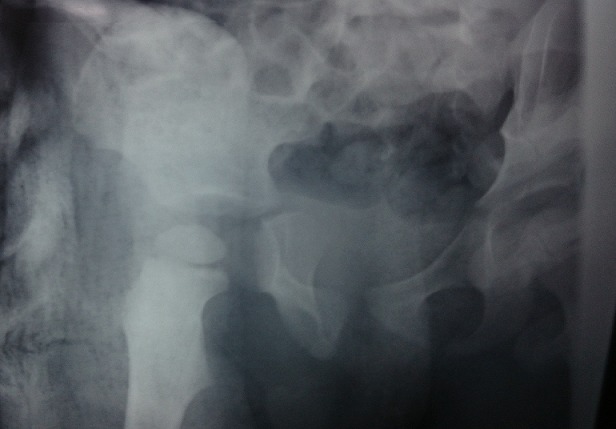
Post-reduction control radiography

**Figure 5:**
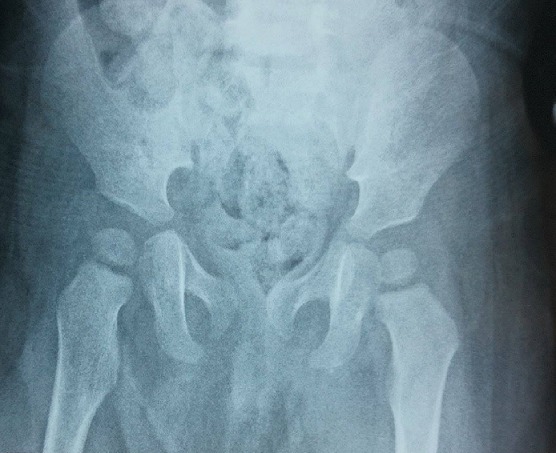
Radiological appearance after 2 years of decline: no evidence of necrosis or changes of the femoral head

A 3 years old boy without any particular diseases history, admitted to the emergency room for a bipolar trauma with a left hemicorporal point of impact following a domestic accident. The delay to consult was 5 hours after the incident. The clinical examination at admission had showed a deformed left superior member, painful on palpation with the presence of the humeral pulse and a right inferior member shortened and flexed, the thigh slightly in adduction, internal rotation with the member. A radiological assessment was performed immediately; including a standard X-ray of the pelvis Figure 1 which showed a dislocated head of the right femur, completed secondarily by a CT Scan of the hip confirming the posterior type of the dislocation isolated from any fractures of the head of the femur Figure 2, Figure 3. The standard radiography of the left shoulder showed a Salter I epiphyseal separation of the humerus. After one hour, we performed an orthopedic reduction of the dislocated hip monitored under scope, with placement of a pelvic splint for an analgesic aim to be kept during three weeks. The radiological results were satisfactory with a restored bilateral symmetry of the articular anatomy Figure 4. The Salter I detachment of the humerus was orthopedically reduced then immobilized by a thoracoabdominal plaster be kept during a month. The child has been discharged the next day. Revised after a month, showed a stable left hip on the clinical examination. In the decline of 2 years, we haven't noticed any recidivism and the functional results were good; On the radiography we do not have any signs of avascular necrosis no changes of the head of femur, of the acetabulum or the joint space Figure 5.

## Discussion

Traumatic dislocation of the hip usually occurs in children after the age of walking, no age predilection. Our child was three years old. The youngest child of the series of Viali and al [3] was two years and four months. Ayadi and al [1] reported a dislocation in a boy of three years. The youngest child in the series of Mehlman and al [4] is aged two years and eight months. Per our patient and most studies [5,6], the male predominance is clear; it is explained perhaps by the most important exhibition of the boys to injury, this contrary to hip dysplasia, where the female sex is predominant. In children under 10 years, the mechanism is minimal: it is mostly a domestic accident, like the case of our patient. This benign trauma contrast with the scarcity of traumatic dislocation of the hip in young children [1]. A factor predisposing to dislocation at this age was found in five patients of the heterogeneous series of Ayadi and al [1], all under six years. This is a moderate coxa valga in four children and a lack of external coverage of the femoral head in moderate four children. However, it seems that these factors only partially explain the benignity of the dislocation at this age since other factors involved were selected by some authors to explain the occurrence of dislocation at this age to know laxity, the capsular ligament fragility, the usual low ages [3,5] as the predominant cartilaginous structure of the acetabulum [7]. However, the dislocation can be idiopathic as the case of our patient. In over 80% of the series in the literature the dislocation is posterior. It is the case of our traumatic dislocation of our patient which was purely of posterior iliac type. Ayadi and al [1] have enumerated 13 cases of posterior dislocation of eight iliac variety and four of ischial variety of 15 hips studied. Brandao [8] objectified 5 cases of pure posterior dislocation of which 3 iliac variety and 2 of ischial variety. According to him, the isolated nature of the dislocation of children under 10 years has been linked to the simple mechanism of dislocation and soft cartilaginous structure of the hip joint at this age, for Brandao, you need a high-trauma energy, which usually occurs in older children, to have the opportunity to observe a femur fracture associated with dislocation [8]. Similarly, Barquet [9] found that the severity of the trauma responsible for the dislocation tends to increase with age.

Most authors are reducing the dislocation under general anesthesia [3,10], others under simple sedation [4,11]. Ayadi and al [1] used general anesthesia in a systematic way at the beginning of their experience, but the finding of the frequency of spontaneous reductions in small children, led to the offer in a child of three years because of the minimal character of the causal trauma to reduce it by a 1 kg stuck weight traction combined with analgesic. A simple muscle relaxation allows the child to the rapid reduction of the dislocation and avoids general anesthesia. In the series of Mehlman and al [4] 62% of children had a reduction simply by sedation. We proceeded to a reduction under general anesthesia for our patient. After the reduction, a radiograph of the pelvis seems sufficient if it is normal [12]. However, the slightest suspicion of a widening spacing is suspect capsular interposition or osteochondral fragment and must move towards the application of computed tomography [3]. In our context, we felt basin radiography of control to be satisfactory. An asset is recommended after the reduction, to reduce pain, allow the absorption of hemarthrosis and the healing of the periarticular soft tissue. There is no consensus in the literature regarding the method or downtime [11,13]. We recommend, if isolated, dislocation in children under six years, immobilization by splint or pelvic-leg cast for four to six weeks and in children over six years pulling stuck for three weeks followed by discharge for another three weeks. Evolution is generally favorable for post-traumatic dislocation of the child. However, a clinical and radiological follow-up is needed for at least 2 years after the trauma. The series showed that there were in 50% of cases, the risk of occurrence of coxa Magna with growth disorders. The complications of traumatic hip dislocations in children are rare compared to those of the adult [**7**]. These complications are also variable depending on age. In small children, the most encountered complications are the interposition of soft parts that may require arthrotomy to extricate [6] and coxa magna by reactive hyperemia to soft tissue injury [3, 7]. This coxa magna, often moderate, has a radiological translation no clinical impact and tends to decrease during growth. No complications were found in our patient seen and had his dislocation reduced in the margin of six hours after his trauma. In older children, complications are more frequent; the most redoubtable is osteonecrosis of the femoral head [2,12]. Other complications is the intra articular incarceration of bone fragments, instability of the hip, heterotopic ossification, premature fusion of the growth plates and post-traumatic osteoarthritis [12,14-16].

## Conclusion

Traumatic dislocations of the hip in children differ from adults by their scarcity, scarcity of associated fractures, their ease of reduction and better prognosis [**7**, **11**]. However, care should be most urgently as possible to overcome the different possible subsequent complications dominated by coxa magna.
